# Dual-Band, Wide-Angle, and High-Capture Efficiency Metasurface for Electromagnetic Energy Harvesting

**DOI:** 10.3390/nano13132015

**Published:** 2023-07-06

**Authors:** Abdulrahman Ahmed Ghaleb Amer, Nurmiza Othman, Syarfa Zahirah Sapuan, Arokiaswami Alphones, Mohd Fahrul Hassan, Ahmed Jamal Abdullah Al-Gburi, Zahriladha Zakaria

**Affiliations:** 1 Faculty of Electrical and Electronic Engineering, Universiti Tun Hussein Onn Malaysia, UTHM, Batu Pahat 86400, Johor, Malaysia; 2 Advanced Sensing Device and Technology FG, Faculty of Electrical and Electronic Engineering, Universiti Tun Hussein Onn Malaysia, UTHM, Batu Pahat 86400, Johor, Malaysia; 3 School of Electrical and Electronic Engineering, Nanyang Technological University, Singapore 639798, Singapore; 4 EMTEX CTS Sdn. Bhd. Incubator Space, Aras 2, Bangunan Penyelidikan, Blok F6, Universiti Tun Hussein Onn Malaysia, Parit Raja, Batu Pahat 86400, Johor, Malaysia; 5 Center for Telecommunication Research & Innovation (CeTRI), Fakulti Teknologi Kejuruteraan Elektrikal dan Elektronik (FTKEE), Universiti Teknikal Malaysia Melaka (UTeM), Ayer Keroh 75450, Malaysia; 6 Centre of Telecommunication Research & Innovation (CeTRI), Fakulti Kejuruteraan Elektronik dan Kejuruteraan Komputer (FKEKK), Universiti Teknikal Malaysia Melaka (UTeM), Durian Tungal 76100, Melaka, Malaysia

**Keywords:** ambient energy harvesting, dual-band, high capture efficiency, metasurface, wide reception angle

## Abstract

A dual-band metasurface (MS) with a wide reception angle operating at Wi-Fi bands (2.4 GHz and 5.4 GHz) is presented for electromagnetic (EM) energy harvesting applications. The MS unit cell comprises a subwavelength circular split ring resonator printed on the low-loss substrate. An air layer is sandwiched between two low-loss substrates to enhance the harvesting efficiency at operating frequencies. One of the main advantages of the proposed MS is that it uses only one harvesting port (via) to channel the captured power to the optimized load (50 Ω), which simplifies the design of a combined power network. According to the results of full-wave EM simulations, the proposed MS has a near-unity efficiency of 97% and 94% at 2.4 GHz and 5.4 GHz, respectively, for capturing the power of incident EM waves with normal incidence. Furthermore, the proposed MS harvester achieves good performance at up to 60° oblique incidence. To validate simulations, the MS harvester with 5 × 5-unit cells is fabricated and tested, and its EM properties are measured, showing good agreement with the simulation results. Because of its high efficiency, the proposed MS harvester is suitable for use in various microwave applications, such as energy harvesting and wireless power transfer.

## 1. Introduction

As low-power devices such as wireless sensors become more widespread, there is a greater demand for sustainable power sources. Because of its potential to increase the mobility and reliability of low-power wireless devices, energy harvesting (EH) has been attracting an abundance of interest. EH is defined as capturing EM power from ambient energy and converting it into usable power [1]. In an EH system, an antenna is the main element used to capture ambient energy, and it desirable for it to have features such as a small size, polarization insensitivity and wide reception angle. Recently, it has become clear that metamaterials offer a promising alternative to traditional antennas, having the main feature of higher efficiency. Metamaterials are defined as an artificial material with unique properties, such as negative permittivity and permeability. With these unique properties, metamaterials can be used in various applications including antennas [2,3], wavefront manipulation [4,5], polarization [6,7,8], and EH [9]. Metamaterials are formed by an array of small electrically resonators arranged in 2D or 3D structures, including split-ring resonators (SRRs) [10] and complementary split-ring resonators (CSRRs) [11,12]. Metamaterials’ resonance allows them to absorb EM power at specific frequencies in the spectrum. In particular, to achieve perfect absorption, it is possible to adjust the material’s effective permittivity and permeability in such a way that the input impedance is well-matched with the free space. However, metamaterial absorbers and harvesters are constructed with the concept of perfect absorption and have significant differences. In the absorbing functionality, most of the absorbed power is dissipated as ohmic or dielectric losses within the structure [13,14,15,16]. In EH applications, a network collects the power harvested by multiple resonators and then delivers it to a rectifier circuit [17,18]. Therefore, one or more grounded resistors are typically loaded within each unit cell in the designs of metamaterial harvesters [19,20]. However, in some recent designs of metamaterial absorbers, the absorbed power is dissipated in lumped resistors located between the resonator’s two sections. Because the resistors in these designs are not grounded, it is difficult to switch them out for a power combining network [21,22,23,24]. The concept of using metamaterial particles as energy collectors for an RF EH was reported in [10]. This indicates that the metamaterial harvesters’ terminals can use resistive loads to export and recycle EM power.

In addition, many types of metamaterial energy harvesters have been developed to improve their applicability, including wide-band [25,26], multi-polarization [27,28], polarization insensitive, wide-incident angle [29,30], and multiband [31,32,33,34]. It is important to note that designs that can capture EM power regardless of the incident wave’s direction improve EH functionality. However, a multiband design enables power collection from a wider variety of radiation sources. The power conversion efficiency is a critical feature of the energy harvester based on metamaterials. To improve the power conversion efficiency, a corporate feed network is used to connect all of the harvester array elements into one load [35,36,37]. A triple-band metasurface resonator with a wide reception angle was designed, with each cell containing four identical SRRs loaded with resistive loads via four harvesting ports (vias) [31]. Harvesting efficiencies of approximately 30%, 90%, and 74% were obtained at 1.75 GHz, 3.8 GHz, and 5.4 GHz, respectively, under normal incidence. In these designs, the power from each cell was split among four loads, making the combining network more complex and less efficient than it needed to be for a practical harvesting system to function. In addition, a metasurface for a wide-angle, triple-band EM EH was developed based on an array of butterfly-shaped closed-ring resonators [38]. A 90%, 83%, and 81% harvesting efficiency was achieved at 0.9 GHz, 2.6 GHz, and 5.7 GHz, respectively. However, the microwave power combining networks’ input impedances were very different from the load resistance needed to achieve such high efficiency (approximately 3 kΩ), limiting the system’s applicability in a real-world harvesting environment. Recently, a symmetric electric-inductive capacitive (ELC) resonator loaded with two edge capacitances and a ring resonator loaded with two resistor loads were used to design a dual-band metasurface resonator [32]. The surface of the metasurface resonator was pixelated, and a binary optimization algorithm was then applied to increase the harvesting efficiency. A 90% harvesting efficiency was reported at 2.45 GHz and 6 GHz. However, it is noted that most multiband metamaterial harvesters have more than one harvesting port (via) for collecting EM energy and delivering it into a load, resulting in a fabrication that is complex, costly, and complicated to build a corporate feed network.

In this study, a novel dual-band metasurface structure with a large reception angle is proposed for EH. The proposed structure consists of a circular resonator printed on a low-loss dielectric substrate. An air layer with a thickness of 1.5 mm is used to improve the harvesting efficiency. A peak harvesting efficiency exceeding 94% is achieved at the operating frequencies of 2.4 and 5.4 GHz under normal incidence. Compared with previous multiband designs with large reception angles [25,32,38], this work is characterized by the following features: Each metasurface unit cell has one port, while in [25,32], each cell is equipped with four and two harvesting ports (via), respectively, which requires more complex power combining networks. Higher efficiency can be achieved with a terminal load of 50 Ω, so the power combining network can be constructed simply. In [38], the terminal load value of 3 kΩ is required.

## 2. Metasurface Unit Cell Design

**Figure 1:**
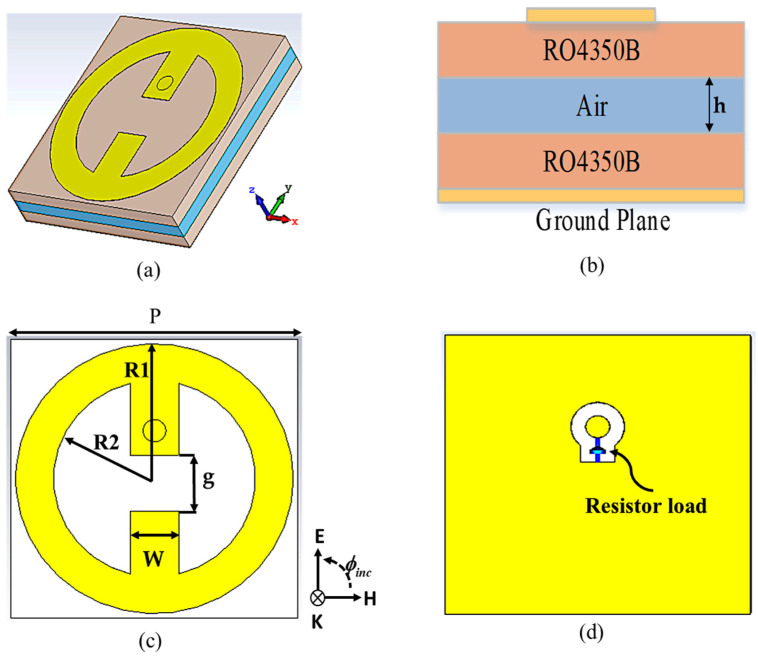
The MS unit cell structure: (**a**) 3D view; (**b**) side view; (**c**) top view; (**d**) bottom view. The dimensions of the MS unit cell were periodicity (P) = 25 mm; outer radius (R1) = 12.1 mm; inner radius (R2) = 8.8 mm; width (W) = 4.2 mm; gap (g) = 5 mm.

For a metasurface harvester, the incident EM waves can be effectively captured at the desired frequencies, and a maximum amount of absorbed power is channeled to a resistor load that models a rectifier circuit. Figure 1 depicts the geometry of the proposed MS harvester. It was designed based on an array of circular split ring resonators operating at dual band frequencies of 2.4 and 5.4 GHz. The proposed MS structure comprises three dielectric layers: top (Rogers), middle (air), and bottom (Rogers). The two face-to-face circular split rings share the same gap printed on the top side of the top dielectric layer. The intermediate dielectric layer is a Rogers RO4350B material with a thickness of 1.524 mm, a dielectric constant of 3.66, and a loss tangent of 0.0037. The metasurface energy harvester can be used to collect energy effectively because of the low energy loss on the dielectric layer. Because of the availability of the harvesting port, a 1.5 mm thick air layer was created to suspend the MS layer. To serve as a ground plane, a copper plate covers the whole back side of the bottom dielectric layer. The top MS resonator and ground plane are made of copper with a thickness of 35 µm and conductivity = 5.8 × 10^−7^ s/m. The incident EM waves is captured by the MS resonator; then, the captured power is delivered to the optimum grounded resistive load through a harvesting port (via). The optimum resistor load value was chosen at 50 Ω, which is suitable for the measurement devices.

CST Microwave Studio was used for the numerical simulation, and periodic boundary conditions were applied along the *x*- and *y*-axes. Floquet ports use two orthogonally polarized plane waves typically propagating in the *x*,*y*-plane to excite the proposed unit cell along the *z*-axis.

The absorptivity A(ω) was calculated using Equation (1):

(1) $$A(\omega )=1-|R(\omega ){|}^{2}-|T(\omega ){|}^{2}$$

whereas R (ω) and T (ω) are the reflection and transmission, respectively. Higher electrical and magnetic losses are necessary for effective absorption of the incident electromagnetic (EM) waves. This can be accomplished by incorporating imaginary components in the relative permittivity and/or permeability. Furthermore, the presence of a metallic layer at the bottom effectively blocks the transmission of the waves so that the transmission can be neglected.

## 3. Results and Analysis

**Figure 2:**
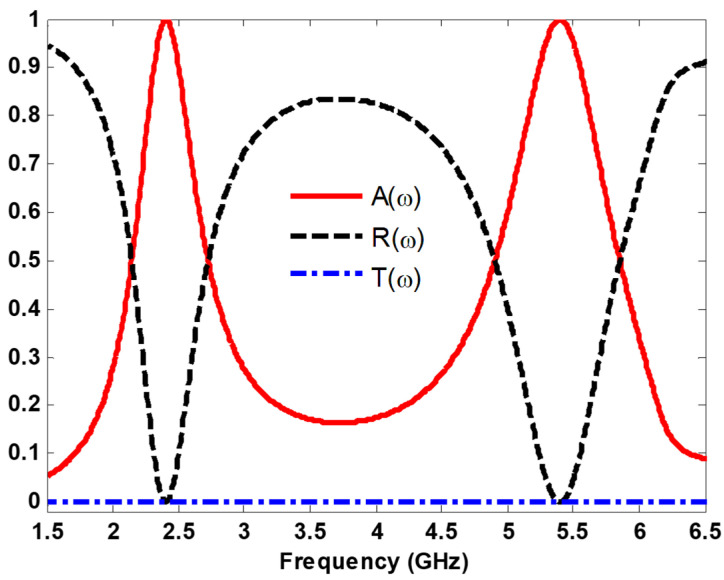
Spectrum of the absorption, reflection, and transmission coefficients.

Figure 2 shows the absorption, reflection, and transmission curves as a function of frequency for the proposed MS harvester at normal incidence. It can be seen that most of the incident power is captured with 99.9% absorption efficiency at 2.4 GHz and 5.4 GHz. In addition, it is clear that the transmission is almost zero because the bottom layer is a full copper plate.

The proposed MS harvester’s design depends on the excitation of magnetic and electric fields. The magnetic field is excited by two currents flowing in opposite directions in the upper and lower metal plates. The top of the MS resonator, where the electric field is concentrated, can be considered as a source of electric excitation. When the electric and magnetic fields occur simultaneously, the absorption can be increased, maximizing the harvesting efficiency.

**Figure 3:**
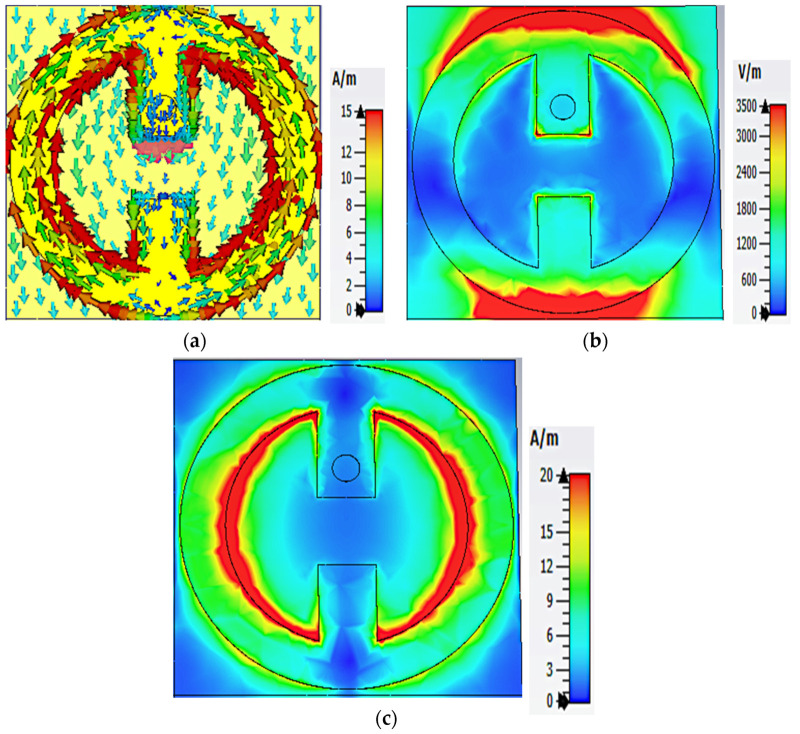
Numerical simulation of (**a**) surface current distribution, (**b**) E-field magnitude, and (**c**) H-field magnitude for the MS array at 2.4 GHz.

**Figure 4:**
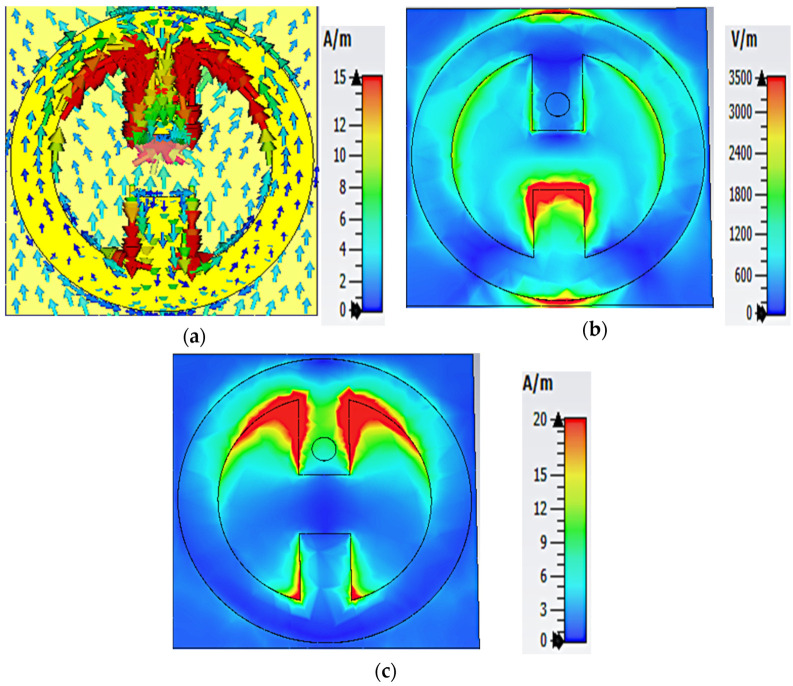
Numerical simulation of (**a**) surface current distribution, (**b**) E-field magnitude, and (**c**) H-field magnitude for the MS array at 5.4 GHz.

To investigate the physical mechanism of collecting EM energy by the proposed MS structure, the distribution of surface current, electric field (E-field), and magnetic field (H-field) at 2.4 GHz and 5.4 GHz were studied, as shown in Figure 3 and Figure 4. Figure 3a shows the surface current distribution for the resonant frequency of 2.4 GHz. It can be seen that the current distribution is more concentrated at the inner side of the circular resonator. Two symmetrical currents are formed at the inner and outer sides of the resonator which flow through the via into the load, resulting in maximum harvesting efficiency. As can be seen in Figure 3b, the E-field dominates at the upper and lower edges of the top MS resonator, while the dominant H-field distribution is enhanced in the inner left and right sides of the upper MS resonator, as shown in Figure 3c. Therefore, most power absorbed at 2.4 GHz is channeled to the load through the via.

Figure 4 illustrates the distribution of the electrical characteristics of the MS array at a frequency of 5.4 GHz. As shown in Figure 4a, an antiparallel current is formed on the resonator’s inner side, increasing absorption. For the E-field the maximum energy is concentrated on the center gap and the outer edges of the resonator along the *y*-axis, while it is less concentrated on the inside of the MS along the *x*-axis, as shown in Figure 4b. From Figure 4c, it can be seen that the maximum energy for the H-field dominates at the top and bottom inner sides of the MS array.

**Figure 5:**
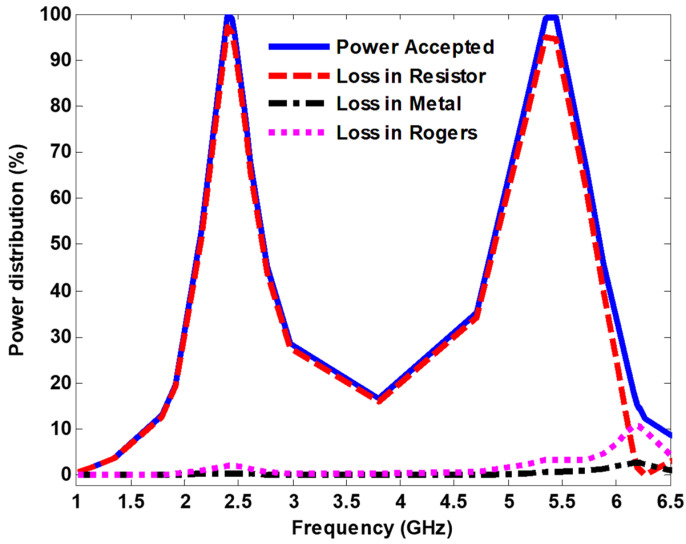
Distribution of the absorbed power into the cell under normal incidence.

The dissipated power distribution into the MS unit cell is investigated and analyzed. Figure 5 depicts the power absorbed by the cell, the harvesting efficiency on the load, substrate and metal under normal incidence, where the incident E-field travel along the *y*-axis (see Figure 1a). The microwave-to-Ac power conversion efficiency can be defined as Equation (2):

(2) $$\eta_{Rad-AC}=\frac{P_{received}}{P_{incident}}$$

where $P_{received}$ represents the power delivered by the load, and $P_{Incident}$ denotes the incident power available on the footprint area. Figure 5 demonstrates that most of the absorbed power is delivered to the load with an efficiency of 97% and 94% at 2.4 GHz and 5.4 GHz, respectively. In addition, since the metal and dielectric have low power dissipation, the incident energy can be collected efficiently.

### 3.1. Oblique Incident Results

**Figure 6:**
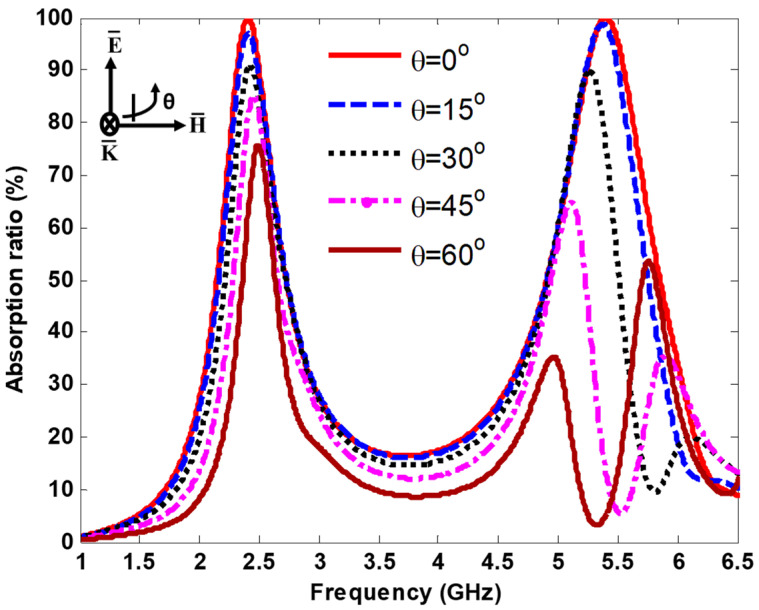
Absorption curves under various incident angles.

At oblique incidence, the reflection coefficient for TE polarized ($\Gamma_{TE}$) can be described as follow:

(3) $$\Gamma_{TE}=\frac{z\;(\omega )\;\cos\;\theta_{i}-z\;(\omega )\;\cos\;\theta_{t}}{z\;(\omega )\;\cos\;\theta_{i}+z\;(\omega )\;\cos\;\theta_{t}}$$

where $\theta_{i}$ and $\theta_{t}$ are the incident angle and transmitted angle, respectively. The absorptivity at different incidence polarization angles for TE polarized was investigated. The TE polarized is defined as the E-field perpendicular to the incident plane, while the H-field is parallel to it. From Equation (3), the reflection coefficient for TE polarization changes when the incident angles ($\theta_{i}$) changes. For TE polarization, the propagation direction of EM wave and direction of the H-field rotate at different incident angles, while the direction of the E-field remains constant. The MS array excitation was studied under different incident and polarization angles to exhibit its ability of capture incoming EM energy, while the direction of the incident EM is random. Figure 6 demonstrates the absorption curves of the MS harvester at various incident angles from 0° to 60° in the step of 15°, under normal incidence. The resonant frequencies shift slightly as the angle of incidence changes. At 2.4 GHz, the absorption ratio, which was more than 90% at 30° and below, w relatively stable. It decreased slightly at θ=45° and 60°, reaching 85% and 76% at 2.45 GHz and 2.49 GHz, respectively. At 5.4 GHz, a near-unity absorption was observed at 15°. In addition, a slight shift was observed when the incident angle changed. At θ=30° and 45°, an absorption response of approximately 90% and 65% was obtained at 5.3 GHz and 5.1 GHz, respectively. When θ=60°, a double absorption peaks of approximately 35% and 54% was observed at 5 GHz and 5.77 GHz, respectively. The absorption peak shifted as the electric field and wave vector effective components changed gradually with oblique incidence. Furthermore, at 5.4 GHz, the additional absorption peaks for TE polarization were visible for large incident angles. As the incident angle increased, the additional absorption curve reached a maximum of 36% and 54% at 45° and 60°, respectively. The additional absorption peaks are caused by parasitic resonances, which increase significantly with incident angle. This enhancement is partially attributed to the angle-dependent higher-order resonances of the interdigitals and calculation errors of the simulation engine in analyzing such a refined structure [39,40].

**Figure 7:**
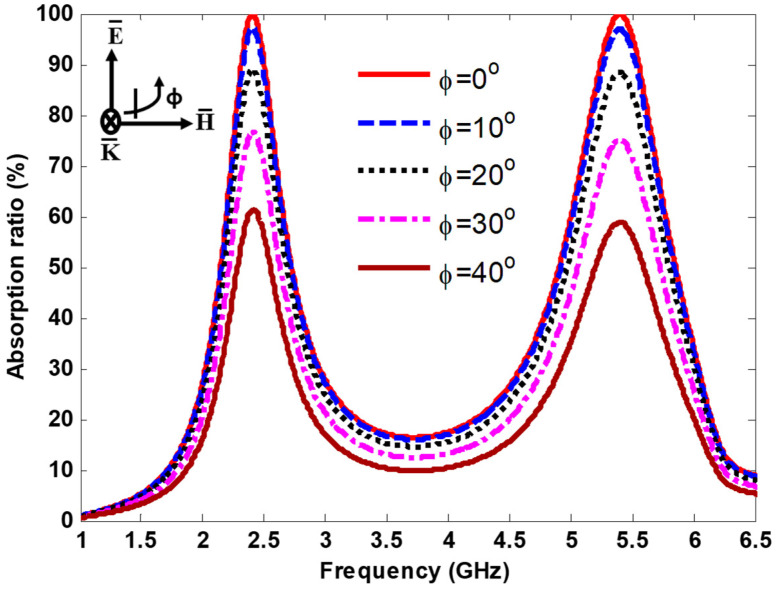
Absorption curves at various polarization angles.

To verify the polarization performance of the proposed MS harvester, the electric and magnetic fields were rotated at different polarization angles with a step of 10, while the propagating direction of the EM wave was kept constant. Figure 7 demonstrates the absorption curves for the MS harvester at various polarization angles (ϕ), from 0° to 40° with a step of 10° under normal incidence. As shown in Figure 7, the MS harvester achieved good absorption for all polarization angles ranging from 0° to 40°. In addition, the absorption behavior decreased as the polarization angles increased because of the decrement of the power incident into the footprint.

**Figure 8:**
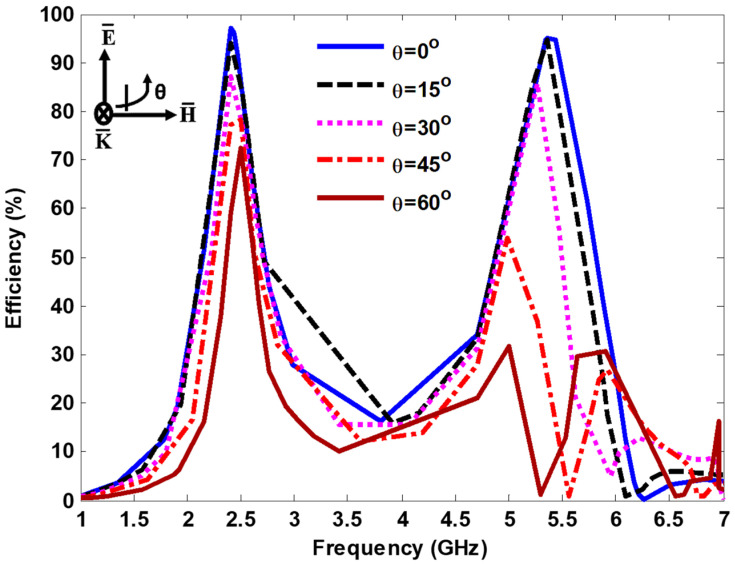
Power efficiency under various incident angles.

Finally, the harvesting efficiency of the proposed MS array was numerically investigated under various incident angles at resonant frequencies using CST Microwave Studio, as shown in Figure 8. At 2.4 GHz, a near unity efficiency of more than 97% and 94% was achieved at θ=0° and 15°, respectively. In addition, a higher harvesting efficiency of approximately 87% was achieved at θ=30°. When the incident angles increased to 45° and 60°, harvesting efficiencies of approximately 77% and 60% were achieved, respectively. At 5.4 GHz, a near unity harvesting efficiency of above 94% was achieved at θ=0° and 15°. As the angle of the incidence increased to θ=30°, a higher harvesting efficiency of approximately 85% was obtained at 5.26 GHz. At θ=45° and 60°, harvesting efficiencies of approximately 54% and 32% were achieved at 5 GHz. Additional absorption peaks were also observed due to the sharp increase in parasitic resonances. As can be seen in Figure 8, the resonant frequency of the harvesting efficiency curves at 2.4 GHz remained constant even when the angle of incidence was oblique. However, the effective components of the electric field and wave vector gradually shifted at 5.4 GHz, resulting in a different peak harvesting efficiency.

### 3.2. Parametric Study

#### 3.2.1. Impact of the Via Hole Position’s Distance from the Center of the Resonator and the Values of the Resistive Load

In the proposed design, some factors played an important role in delivering the maximum amount of absorbed power to the resistive load, improving the harvesting efficiency. First, the use of a low-loss dielectric substrate allows most of the absorbed power to be directed from surface of the resonator to the resistive load rather than being dissipated in a lossy substrate such as the metamaterial absorber. In addition, the optimal value of the resistive load that achieves a good impedance match between the input impedance of the MS structure, and the free space allows the maximum power to be transferred from the top layer of the resonator to the load, improving the harvesting efficiency.

**Figure 9:**
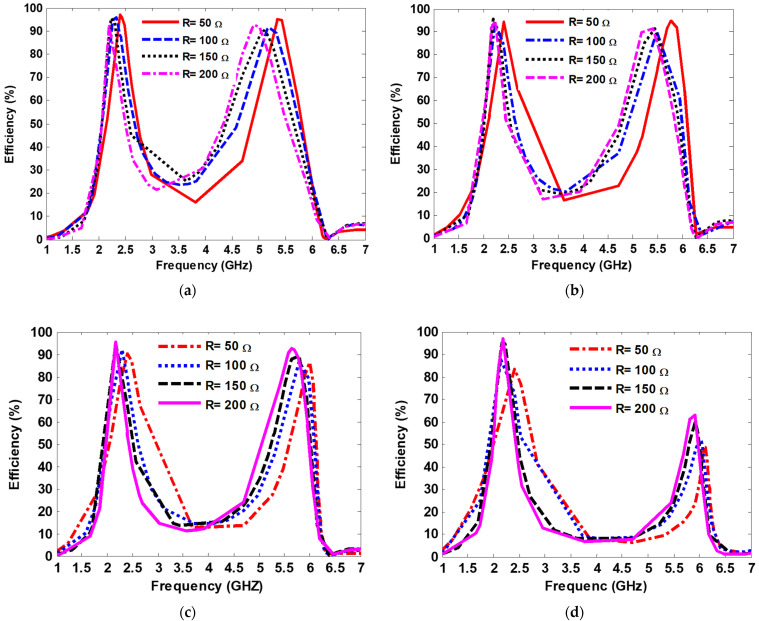
Numerical results showing the harvesting efficiency of the proposed MS harvester with various terminated resistive loads ranging from R = 50 Ω to R = 200 Ω, and having a via position distance away from the center of (**a**) 4.3 mm; (**b**) 5.9 mm; (**c**) 7.5 mm; (**d**) 9.1 mm.

In order to examine the influence of the via hole position relative to the resonator’s center on harvesting efficiency, the via hole position was varied from 4.3 mm to 9.1 mm in increments of 1.6 mm. The terminated resistive load was swept from R = 50 to R = 200 in four cases of via positions. Then, the harvesting efficiency of the proposed MS harvester was computed. Figure 9 shows the numerical results showing the harvesting efficiency of the proposed MS harvester with various terminated resistive loads ranging from R = 50 Ω to R = 200 Ω and with various via position distance away from the center of the resonator. The harvesting efficiency peaks of 97% and 94% at 2.4 GHz and 5.4 GHz, respectively, were achieved when the MS harvester was terminated by a resistive load R = 50 Ω, and the via position distance away from the center of the resonator was 4.3 mm. Furthermore, as observed in Figure 9, the harvesting efficiency of the proposed MS harvester at 2.4 GHz remained stable for different via positions when terminated by a resistive load of R = 50 Ω. However, at the resonant frequency of 5.4 GHz, the harvesting efficiency decreased with an increment of the via position, as depicted in Figure 9d.

#### 3.2.2. Effect of the Inner Radius (R2)

**Figure 10:**
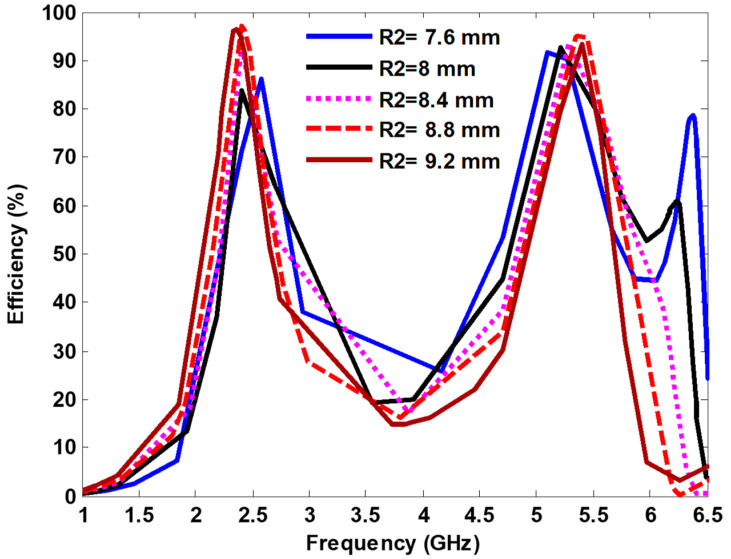
Efficiency curves for different circular inner radii.

Figure 10 shows the harvesting efficiency of the proposed MS harvester by changing the inner radius (*R2*) of the circular resonator from 7.6 mm to 9.2 mm, and it is observed that the harvesting efficiency increased while increasing the *R2*. Furthermore, the harvesting efficiency remained stable at 2.4 GHz, and it shifted at the higher side at 5.4 GHz while the *R2* decreased. The maximum harvesting efficiencies for the both resonance frequencies were achieved at *R2* = 8.8 mm.

#### 3.2.3. Effect of the Air Layer

**Figure 11:**
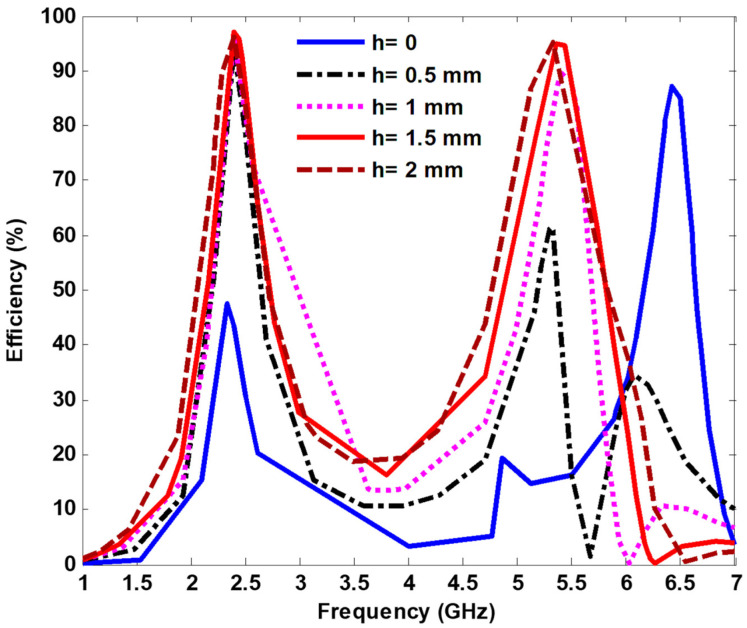
Efficiency with different air layer thicknesses.

Figure 11 shows the harvesting efficiency for the proposed MS harvester with different air layer thicknesses (h), and it was observed that the harvesting efficiency increased with an increase of the air layer thicknesses. Because of the availability of the harvesting port, an air thickness of 1.5 mm was selected. The harvesting efficiency peak exceeding 97% and 94% was achieved for 2.4 GHz and 5.4 GHz, respectively. By adding the air layer, the matching impedance between the MS harvester and terminated load improved, enhancing the harvesting efficiency.

## 4. Measurement Verification and Discussion

**Figure 12:**
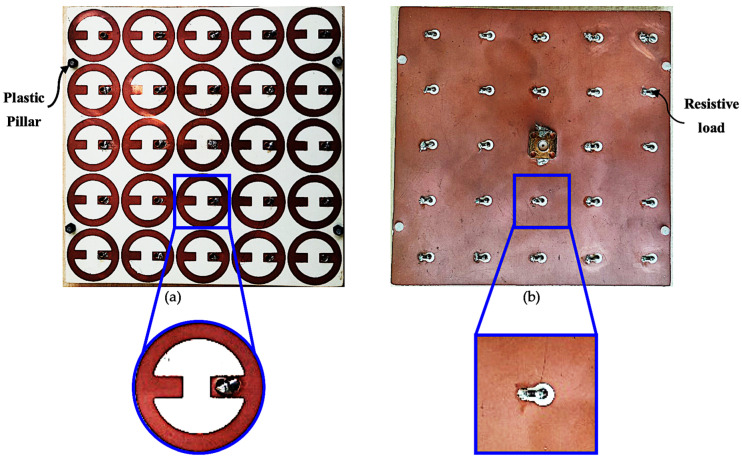
Photo of the fabricated MS harvester array: (**a**) top side; (**b**) bottom side.

To compare the simulation and measurement results, an experimental study was conducted. The fabricated harvester consists of a 5 × 5 MS unit cell array printed on a Rogers RO4350B substrate, as shown in Figure 12. There were four dielectric pillars connecting and supporting the top and bottom Rogers RO4350B materials. To increase the efficiency and match the input impedance of the MS array to the measurement equipment, a 1.5 mm thick air layer was sandwiched between dielectric substrates. The overall dimensions of the proposed MS array were 125 mm × 125 mm. Each MS resonator was connected to the ground plane using optimal resistive load (50 Ω) through a metallic via, except the central unit cell, which was connected to the SMA connector. Since the central cell resembles a simulation of an infinite array, it was selected for measurement.

**Figure 13:**
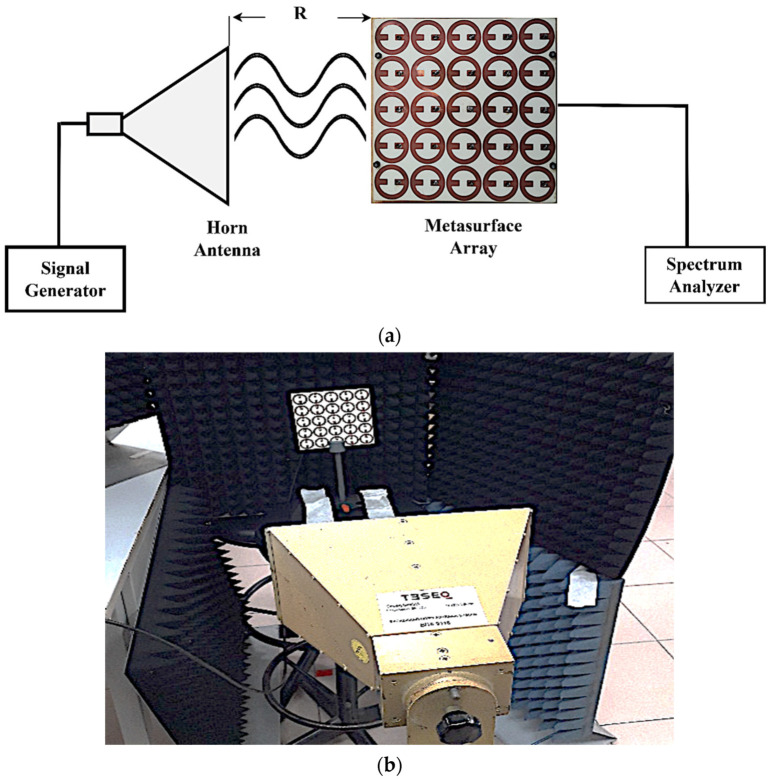
(**a**) Schematic of the measurement set-up; (**b**) actual measurement set-up.

Figure 13 shows the measurement set-up for the power received from the central unit cell. The fabricated MS array was placed in the far-field region of the standard horn antenna. The standard horn antenna covers the measurement band (1–7 GHz) and was fed with the RF signal was generated by a signal generator (Agilent Technologies CXA, Santa Clara, CA, USA). The central cell of the MS array was connected to a spectrum analyzer (Agilent Technologies E8267D) to measure the power received by the cell.

The overall efficiency of the MS array can be determined using Equation (2). The incident power absorbed in the surface region of the MS array can be described as Equation (4)

(4) $$\;P_{incident}=\frac{G_{t}P_{t}}{4\pi R^{2}}\times A_{\left(eff,array\right)}$$

where $G_{t}$ is the horn antenna’s gain, $P_{t}$ is the signal generator’s excitation power, and R is the horn antenna-to-prototype distance. Furthermore, $A_{\left(eff,\;array\right)}=25\;A_{eff}$ is the MS array’s effective area, which refers to the number of unit cells, and $A_{eff}$ is the effective area of the central unit cell [41].

**Figure 14:**
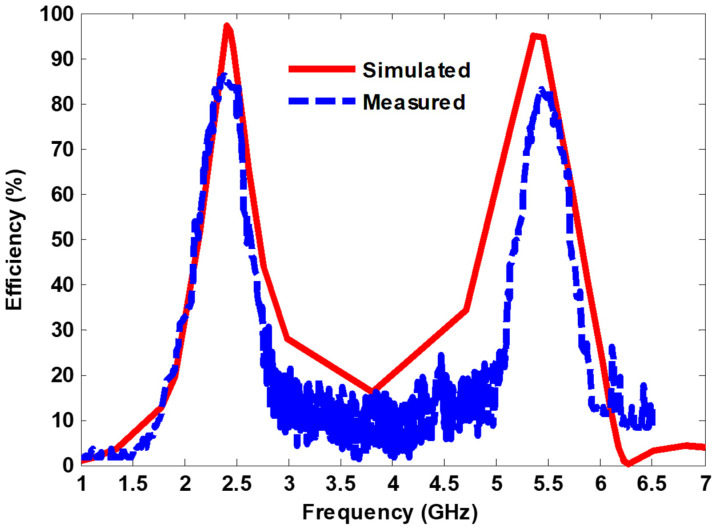
Simulated and measured harvesting efficiency under normal incidence.

Figure 14 shows the simulated and measured harvesting efficiency at normal incidence when the E-field is parallel to the *y*-axis. It can be seen that the measured efficiency of approximately 85% and 83% was achieved at 2.38 GHz and 5.43 GHz, respectively. In addition, a good agreement between the simulation and measurement results could be observed.

Table 1 shows the advantages of the proposed MS harvester presented in this research compared with the previously published dual-band and multiband MS harvester works. It can be seen that the proposed MS harvester can efficiently capture the EM power and then deliver it to the optimal resistive load using one via/harvesting port, achieving higher harvesting efficiency at the operating frequency bands. Compared with other related works, the proposed MS harvester uses one via/harvesting port, which makes it simpler and reduces the complexity of building the power combining network to connect all array elements in a single load, increasing the overall harvesting efficiency.

## 5. Conclusions

This research proposes a novel dual-band, wide-reception angle metasurface that captures EM power with high harvesting efficiency while simplifying the structure. The simulation results demonstrate that a periodic configuration of the proposed metasurface resonators with an optimized resistive load (50 Ω) can absorb 99.9% of a normal incident wave at 2.4 GHz and 5.4 GHz. Furthermore, the most absorbed power delivers to the load through one harvesting port (via) resulting in an impressive efficiency exceeding 97% and 94% at 2.4 GHz and 5.4 GHz, respectively. For the proof of concept, an array of 5 × 5 cells was fabricated using a printed circuit board and experimentally evaluated. The measured and simulated results agreed well. It should be noted that, unlike the multiband designs of earlier works, the presented metasurface only needs a single via to deliver the absorbed power to a resistive load with a low impedance value, making it more practical for use in practical harvesting systems.

## Figures and Tables

**Table 1:** Comparison of the proposed MS harvester with other published related works.

Ref.	Frequency Bands (GHz)	Unit Cell Size	Substrate Material	No. of Via	Efficiency		Complexity to Build Power Combining Network
					Simulated	Measured	
[25]	7, 15, 19.5 (triple-band)	0.63 λ	F4B-2	Four	Above 90%	82.3%, 90.7%, and 88.7%	Complex
[31]	1.75, 3.8, and 5.4 (triple-band)	0.57 λ	Rogers RO4003	Four	30%, 90%, and 74%	26%, 88%, and 72%	Complex
[32]	2.45 and 6 (dual-band)	0.23 λ	Rogers RT/duroid 6006	Two	95% and 90%	90% and 85%	Complex
[38]	0.9, 2.6, and 5.7 (triple-band)	0.51 λ	F4B	Two	90%, 83%, and 81%	65%, 70%, and 70%	Complex
[42]	5.5 and 7.2 (dual-band)	0.15 λ	Rogers RO4003C	Four	94% and 93%	None	Complex
[43]	2.9 and 5.1 (dual-band)	0.14 λ	Rogers RT6006	Two	85% and 45%	None	Complex
This work	2.4 and 5.4 (dual-band)	0.45 λ	Rogers RO4350B	One	97% and 94%	85% and 83%	Simple
